# Abdominal Cystic-Like Lesion as a Rare Complication of Neglected Infectious Disease

**DOI:** 10.1371/journal.pntd.0004947

**Published:** 2016-09-29

**Authors:** Priscila Marques de Macedo, Rodrigo Almeida-Paes, Dayvison Francis Saraiva Freitas, Paula Marsillac, Ana Paola de Oliveira, Flavia Antelo Saez, Bodo Wanke, Antonio Carlos Francesconi do Valle

**Affiliations:** 1 Infectious Dermatology Clinical Research Laboratory, Evandro Chagas National Institute of Infectious Diseases, Fiocruz, Rio de Janeiro, Brazil; 2 Mycology Laboratory, Evandro Chagas National Institute of Infectious Diseases, Fiocruz, Rio de Janeiro, Brazil; 3 Department of Internal Medicine, Bonsucesso Federal Hospital, Rio de Janeiro, Brazil; 4 Radiology Department, Evandro Chagas National Institute of Infectious Diseases, Fiocruz, Rio de Janeiro, Brazil; University of California San Diego School of Medicine, UNITED STATES

## Case Discussion and Question

**Fig 1:**
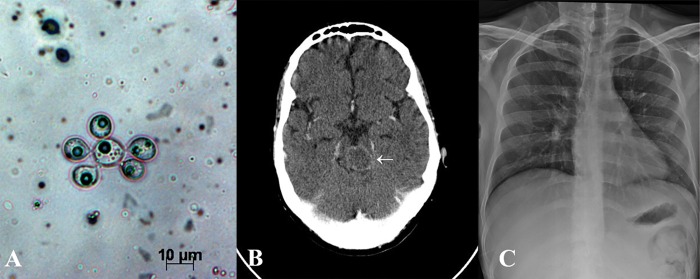
Laboratory and imaging results of the patient with acute paracoccidioidomycosis. **A.** *Paracoccidioides* spp. multiple budding cells in KOH preparation from the skin biopsy. **B.** Brain CT scan showing a pontomesencephalic tumor lesion (2.3 cm) with ring-enhancement after intravenous contrast (white arrow). **C.** Normal chest X-ray.

An 18-year-old, HIV-negative, male patient was admitted to the hospital complaining of nonquantified weight loss and abdominal pain over a 3 month period. Physical findings included disseminated cutaneous lesions and paraparesis of the lower extremities. Diagnosis of acute paracoccidioidomycosis was confirmed through KOH (potassium hydroxide) direct visualization of typical yeast structures of *Paracoccidioides* spp. in samples from the skin biopsy (Fig 1A). Abdominal ultrasound revealed extensive peritoneal lymph node involvement, and computerized tomography (CT) with intravenous contrast of the brain revealed a pontomesencephalic lesion (2.3 cm) with ring-enhancement (Fig 1B, white arrow). Chest radiography was normal (Fig 1C).

**Fig 2:**
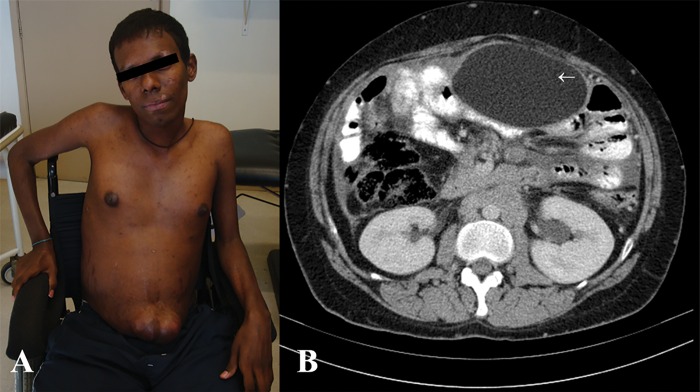
**A.** Patient’s abdominal bloating with a large umbilical hernia and scarring lesions in the face and trunk. **B.** Abdominal CT scan with oral and intravenous contrast reveals an 11.1 x 6.5 cm lesion with a density consistent with fat (-56 HU) in the anterior wall of the abdominal cavity (white arrow).

Liposomal amphotericin B was administered for 90 days, followed by sulfamethoxazole/trimethoprim. The patient responded well to therapy, but after approximately 1 year of drug therapy, he developed painful abdominal bloating and umbilical protrusion (Fig 2A). Abdominal CT with oral and intravenous contrast revealed a 11.1 by 6.5 cm ovoid lesion in the anterior abdominal wall (Fig 2B, white arrow) that was previously reported as a similar cystic image by ultrasound a month before.

What is your diagnosis?

## Answer and Discussion

**Chylous ascites** is the extravasation of chyle into the peritoneal cavity, and this condition is defined as the presence of more than 110 mg/dL triglyceride levels in the ascitic fluid [1]. Any source of lymph vessels obstruction can cause chylous effusion. It is an uncommon type of ascites, frequently related with malignant conditions (lymphomas and peritoneal metastasis) as well as intra-abdominal surgery. Infectious diseases such as lymphatic filariasis, ascaridiasis, peritoneal tuberculosis, and *Mycobacterium avium* infection in HIV patients are rare conditions that can cause chyloperitoneum [2,3].

Acute paracoccidioidomycosis (PCM) is a potential life-threatening neglected systemic mycosis endemic to Latin America that mostly affects lymph abdominal and mononuclear phagocytic organs of young, vulnerable patients [4]. Chylous ascites secondarily to PCM is considered a severe manifestation of the mycoses, and it is extremely uncommon. In the case herein presented, as a result of extensive inflammatory damage to the patient’s abdominal lymph nodes, combined with the fibrotic scarring that occurred during the healing process, caused the obstruction of the lymphatics, resulting in the leakage of chyle into the abdominal cavity. As a consequence, a rounded image formation occurred that was initially noted as a cystic-like image by ultrasound and subsequently correctly diagnosed by CT imaging as loculated ascites with an increased density due to the accumulation of fat. Laboratory analyses of samples obtained by ultrasound-guided paracentesis of the ascitic fluid within the lesion revealed high levels of triglycerides (5,200 mg/dL), and cultures for bacteria, mycobacteria, and fungi were all negative. The high level of triglycerides and the absence of microorganisms confirmed the diagnosis of pure chylous ascites. The first step in the treatment of chylous ascites includes the addition of low lipid, high medium-chain triglycerides to the patient’s diet followed by parenteral nutrition only if oral supplementation fails [5]. In addition, serial paracenteses can be indicated to reduce intra-abdominal pressure and also to relieve symptoms. Somatostatin analogues can also be administered in order to reduce lymphorragia. Surgical approaches are only indicated if clinical therapy has failed due to its high morbidity; surgical methods include direct lymph vessels ligation and peritoneal-venous shunting [5]. In this case, the patient’s symptoms were initially managed with nutritional measures, albeit surgical excision of the lesion is under consideration (Box 1).

**Box 1. Key Learning Points**

- Chylous ascites may be a severe complication of neglected infectious diseases, including paracoccidioidomycosis.
- Early diagnosis and treatment of this systemic mycosis can reduce the inflammatory damage to the lymphatic system and prevent this severe outcome.
- Analysis of ascitic fluid is essential to distinguish chylous ascites from active infectious diseases, such as a bacterial or fungal abscess.

## Ethics Statements

The Research Ethics Committee of Evandro Chagas National Institute of Infectious Diseases (INI)/Fiocruz has approved this study protocol under the register CAAE 42590515.0.0000.5262. The patient has signed the informed written consent form for this publication.
